# Metabolic Profiles in Cell Lines Infected with Classical Swine Fever Virus

**DOI:** 10.3389/fmicb.2017.00691

**Published:** 2017-04-20

**Authors:** Hongchao Gou, Mingqiu Zhao, Jin Yuan, Hailuan Xu, Hongxing Ding, Jinding Chen

**Affiliations:** College of Veterinary Medicine, South China Agricultural UniversityGuangzhou, China

**Keywords:** metabolomics, CSFV, PK-15 cells, 3D4/2, classical swine fever virus

## Abstract

Viruses require energy and biosynthetic precursors from host cells for replication. An understanding of the metabolic interplay between classical swine fever virus (CSFV) and host cells is important for exploring the complex pathological mechanisms of classical swine fever (CSF). In the current study, and for the first time, we utilized an approach involving gas chromatography coupled with mass spectrometry (GC-MS) to examine the metabolic profiles within PK-15 and 3D4/2 cells infected with CSFV. The differential metabolites of PK-15 cells caused by CSFV infection mainly included the decreased levels of glucose 6-phosphate [fold change (FC) = −1.94)] and glyceraldehyde-3-phosphate (FC = −1.83) during glycolysis, ribulose 5-phosphate (FC = −1.51) in the pentose phosphate pathway, guanosine (FC = −1.24) and inosine (FC = −1.16) during purine biosynthesis, but the increased levels of 2-ketoisovaleric acid (FC = 0.63) during the citrate cycle, and ornithine (FC = 0.56) and proline (FC = 0.62) during arginine and proline metabolism. However, metabolite changes caused by CSFV infection in 3D4/2 cells included the reduced glyceraldehyde-3-phosphate (FC = −0.77) and pyruvic acid (FC = −1.42) during glycolysis, 2-ketoglutaric acid (FC = −1.52) in the citrate cycle, and the elevated cytosine (FC = 2.15) during pyrimidine metabolism. Our data showed that CSFV might rebuild cellular metabolic programs, thus aiding viral replication. These findings may be important in developing targets for new biomarkers for the diagnosis and identification of enzyme inhibitors or metabolites as antiviral drugs, or screening viral gene products as vaccines.

## Introduction

Classical swine fever virus (CSFV) belongs to the *Flaviviridae* family, and is related to hepatitis C and dengue virus (Paton et al., 1995; Becher et al., 2003). The single-stranded positive RNA of CSFV contains a unique large open reading frame (ORF) which encodes a polyprotein subsequently processed into 12 known proteins by cellular and viral proteases (Thiel et al., 1991; Moennig and Plagemann, 1992). Classical swine fever (CSF) of piglets, caused by CSFV infection, is characterized by hemorrhagic syndrome and immunosuppression (Susa et al., 1992; Summerfield et al., 1998). Because of its high morbidity and mortality, CSF is A-listed by the OIE (World Organization for Animal Health) (Paton and Greiser-Wilke, 2003). Although CSF is an important disease for animals worldwide, its eradication is difficult because there has been little recent study of its molecular mechanisms (Lange et al., 2011; Blome et al., 2013).

To explore the complex interaction between CSFV and host cells, genomic and proteomic approaches have been employed to analyze the relevant cellular mechanisms (Sun et al., 2008; Li J. et al., 2010; Li S. et al., 2010). Compared to genomics and proteomics, metabolomics is a biological approach providing top-down insights into the systemic pattern of low molecular weight compounds rather than functional intermediates (Fiehn, 2002). Previous reports of metabolomics show great promise in the evaluation of viral infection mechanisms (Munger et al., 2006; Birungi et al., 2010; Vastag et al., 2011). However, systematic changes in metabolites in CSFV-infected cells remain unknown.

It is particularly important to select reasonable cell models for exploring the metabolic profiles of viral replication (Aldridge and Rhee, 2014). The PK-15 cell line is usually used to research CSFV replication and maturation *in vitro* (Grummer et al., 2006), while 3D4/2 is a macrophage cell line closely related to monocytic cells, which are target cells for CSFV infection *in vivo* (Lange et al., 2011). In the current study, both PK-15 and 3D4/2 cells infected by CSFV (Shimen strain) were analyzed using a metabolomics platform based on gas chromatography coupled with mass spectrometry (GC-MS). The metabolic changes of CSFV-infected cells were predicted via MetaboAnalyst 2.0. The results showed that glycolysis, the citrate cycle, amino acid metabolism, nucleotide biosynthesis, and lipid metabolism in PK-15 and 3D4/2 cells were utilized by CSFV to improve the rate of viral infection. The current study provides the first data regarding the regulation of the metabolic network in CSFV-infected cells.

## Materials and methods

### Reagents and antibodies

Dulcitol and methoxylamine hydrochloride were purchased from Sigma (Sigma–Aldrich, USA). Methanol, pyridine, and BSTFA (1% TMCS) were purchased from ANPEL (ANPEL, China). Antibodies including Mouse monoclonal anti-CSFV E2 (WH303) (JBT, 9011) and Dylight 488 goat anti-mouse IgG (EarthOx, E032210) were used for indirect immunofluorescence.

### Cell culture and virus

The swine kidney cell line PK-15 (ATCC, CCL-33) and porcine macrophage cell line 3D4/2 (ATCC, CRL-2845) were cultured as described previously (Pei et al., 2014). The CSFV strain (Shimen) used in the study was prepared as described previously (Pei et al., 2014).

### Virus titration

To measure viral titers, cells cultivated in 96-well plates were infected with 10-fold serial dilutions of CSFV. After culture at 37°C for 48 h, cells were fixed with 80% cold acetone diluted with phosphate-buffered saline (PBS) at −20°C for 30 min. Virus infection was detected by indirect immunofluorescence assay using the CSFV E2 protein-specific monoclonal antibody WH303 (Lin et al., 2000), followed by Alexa 488-conjugated goat anti-mouse IgG. Virus titers were calculated according to Kaerber and expressed as 50% tissue culture infectious doses (TCID_50_) per milliliter.

### Viral infection

PK-15 or 3D4/2 cells with 80% confluence were infected with CSFV at a multiplicity of infection (MOI) of 5. MOI was evaluated according to the virus titer from the respective cell line. The mock group was infected with an equal volume of Dulbecco's modified Eagle's medium (DMEM). After 1 h of aspiration, the inoculum was aspirated and then washed twice with PBS. Cells were subsequently incubated in DMEM or RPMI 1640 medium containing 10% FBS and 1% antibiotics at 37°C for a different number of hours post infection (hpi).

### Virus one-step growth curve

PK-15 or 3D4/2 cells with 80% confluence in a 75 cm^2^ cell culture flask were infected with CSFV at an MOI of 5. Subsequently, 100 μL of viral supernatant was, respectively, harvested at 12, 24, 36, 48, 60, 72, 84, and 96 hpi. The virus one-step growth curve was drafted according to viral titers.

### Sample preparation

At 24 h before CSFV infection, ~2 × 10^5^ PK-15 or 3D4/2 cells were seeded in each well of 6-well cell culture plates to obtain 80–90% confluence. For each cell line, two 6-well plates were respectively prepared. One 6-well plate was infected by CSFV at an MOI of 5, while the other was used as the mock group. All cells (about 1.6 × 10^6^) in each well were harvested at 48 hpi. Briefly, after medium was discarded, the cells were washed twice with cold PBS, quenched with 400 μL of cold methanol (−80°C) and placed at −80°C for 30 min. Another 400 μL of pure water was added and cells were scraped off the plates. The mixture was transferred for metabolomics sample preparation.

### Metabolite extraction and derivatization

The mixture was sonicated in an ice bath for 5 min and placed at −20°C for 24 h before centrifugation at 16,000 g for 15 min at 4°C. In total, 400 μL of supernatant and 10 μL of internal standards (0.1 mg/mL dulcitol) were mixed and evaporated until dry under a gentle nitrogen stream. The residues were reconstituted in 30 μL of 20 mg/mL methoxyamine hydrochloride in pyridine, and incubated at 37°C for 90 min. Then 30 μL of BSTFA (with 1% TMCS) was spiked and derivatization was performed at 70°C for 60 min prior to GC-MS analysis.

### GC-MS analysis

Analysis of metabolites was performed on an Agilent 7890A/5975C GC-MS system (Agilent Technologies, USA). The derivatives were separated using an HP-5MS fused-silica capillary column (30 m × 0.25 mm × 0.25 μm, Agilent J&W Scientific, USA) with helium (>99.999%) as a carrier gas at a constant flow rate of 1 mL/min. Injection volume was 1.5 μL in splitless mode, and the solvent delay time was 6 min. The initial oven temperature was 70°C for 2 min, ramped to 160°C at a rate of 6°C/min, to 240°C at a rate of 10°C/min, to 300°C at a rate of 20°C/min, and finally held at 300°C for 6 min. The temperatures of injector, transfer line, and electron impact ion source were set to 250°C, 290°C, and 230°C, respectively. The electron energy was 70 eV, and data was collected in a full scan mode (m/z 50-600).

### Data preprocessing and statistical analysis

The baseline filtering, peak finding, alignment, deconvolution, and further processing of raw GC-MS data were carried out according to previous protocols (Gao et al., 2010). The final data was exported as a peak table file, including observations (sample name), variables (rt_mz), and peak intensities. The data was normalized against total peak intensities before performing univariate and multivariate statistics. The peak table (named matrix X) file was imported to Simca-P (version 11.0, Umetrics AB, Sweden), where multivariate statistical analyses, such as PCA, PLS-DA, and OPLS-DA, were performed. All data were mean-centered and unit variance (UV)-scaled prior to multivariate statistical analysis. The quality of the models is described by the R^2^X or R^2^Y and Q^2^ values.

### Identification and structural validation of differential metabolites

Differential metabolites were determined by the combination of the variable importance in the projection (VIP) value (>1) of the OPLS-DA model and the *p*-values (<0.05) from two-tailed Student's *t*-tests on normalized peak intensities. FC was calculated as a binary logarithm of average normalized peak intensity ratio between the CSFV-infected and mock groups, where the positive value indicates that the average mass response of CSFV-infection is higher than the mock group. The structural identification of differential metabolites was performed with AMDIS software using automatic matching with an in-house standard library including retention time and mass spectra, Golm Metabolome Database, and Agilent Fiehn GC/MS Metabolomics RTL Library.

### Metabolic pathway analysis

Metabolic pathway analysis was performed via MetaboAnalyst2.0 (http://www.metaboanalyst.ca/MetaboAnalyst/).

## Results

### Characteristics of PK-15 and 3D4/2 cells infected by CSFV

**Figure 1:**
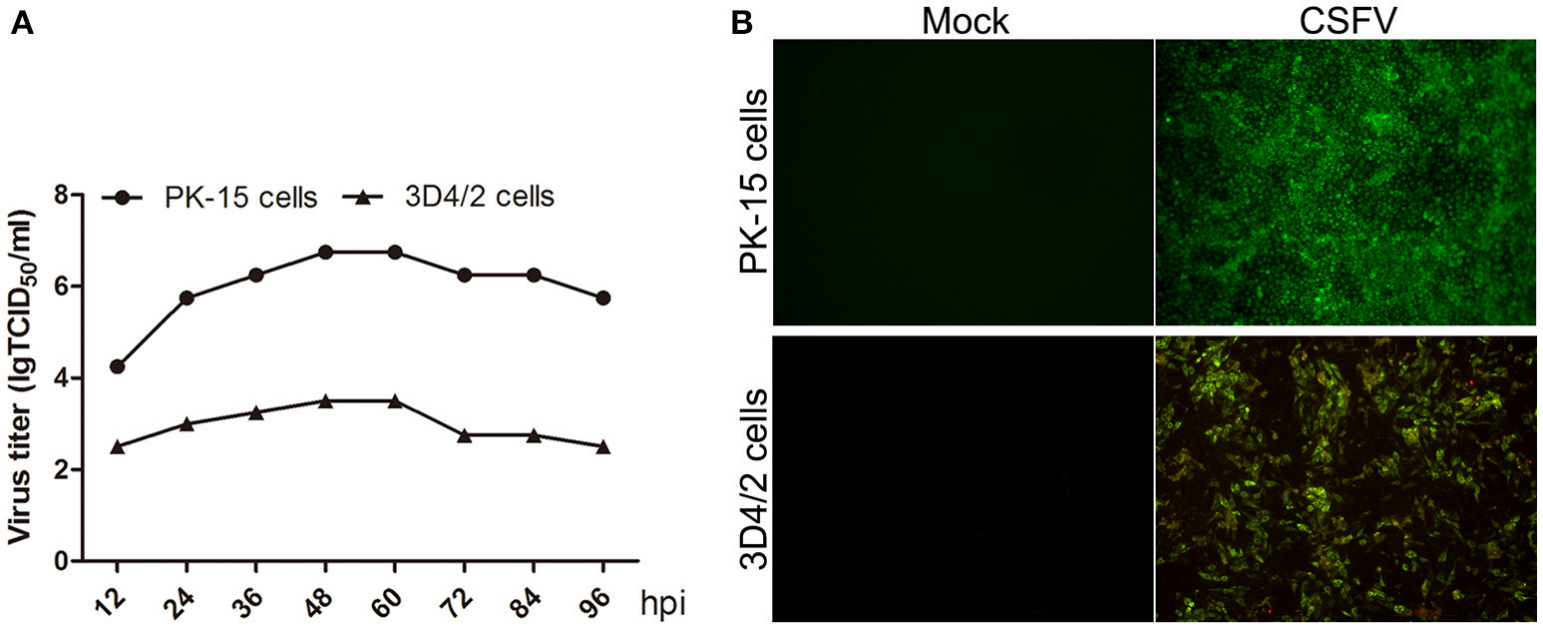
**(A)** Growth curves of CSFV in PK-15 and 3D4/2 cells. PK-15 and 3D4/2 cells were infected by CSFV (MOI = 5), and the viral once growth curve was determined as described in Materials and Methods. **(B)** Infection rates of PK-15 and 3D4/2 cells by CSFV at 48 hpi. PK-15 and 3D4/2 cells were infected by CSFV (MOI = 5), and the rates of cells infected by CSFV were observed by using immunofluorescence assay as described in materials and methods. The images were snapped by immunofluorescence microscope under the 20 × objective.

CSFV does not cause cytopathologic effects (CPE) in both PK-15 and 3D4/2 cells (data not shown). To analyze CSFV replication in PK-15 and 3D4/2 cells, virus one-step growth curve was determined by measuring viral titers using an indirect immunofluorescence assay. As shown in Figure 1A, virus titers in PK-15 and 3D4/2 cells both reached peak value at 48 h post infection (hpi). Immunofluorescence microscopic observation showed that >90% PK-15 and 3D4/2 cells were infected with CSFV at 48 hpi (Figure 1B). These results suggested that mock- or CSFV-infected PK-15 and 3D4/2 cells should be prepared at 48 hpi for analysis of metabolomics.

### Analysis of metabolomics based on GC-MS

**Figure 2:**
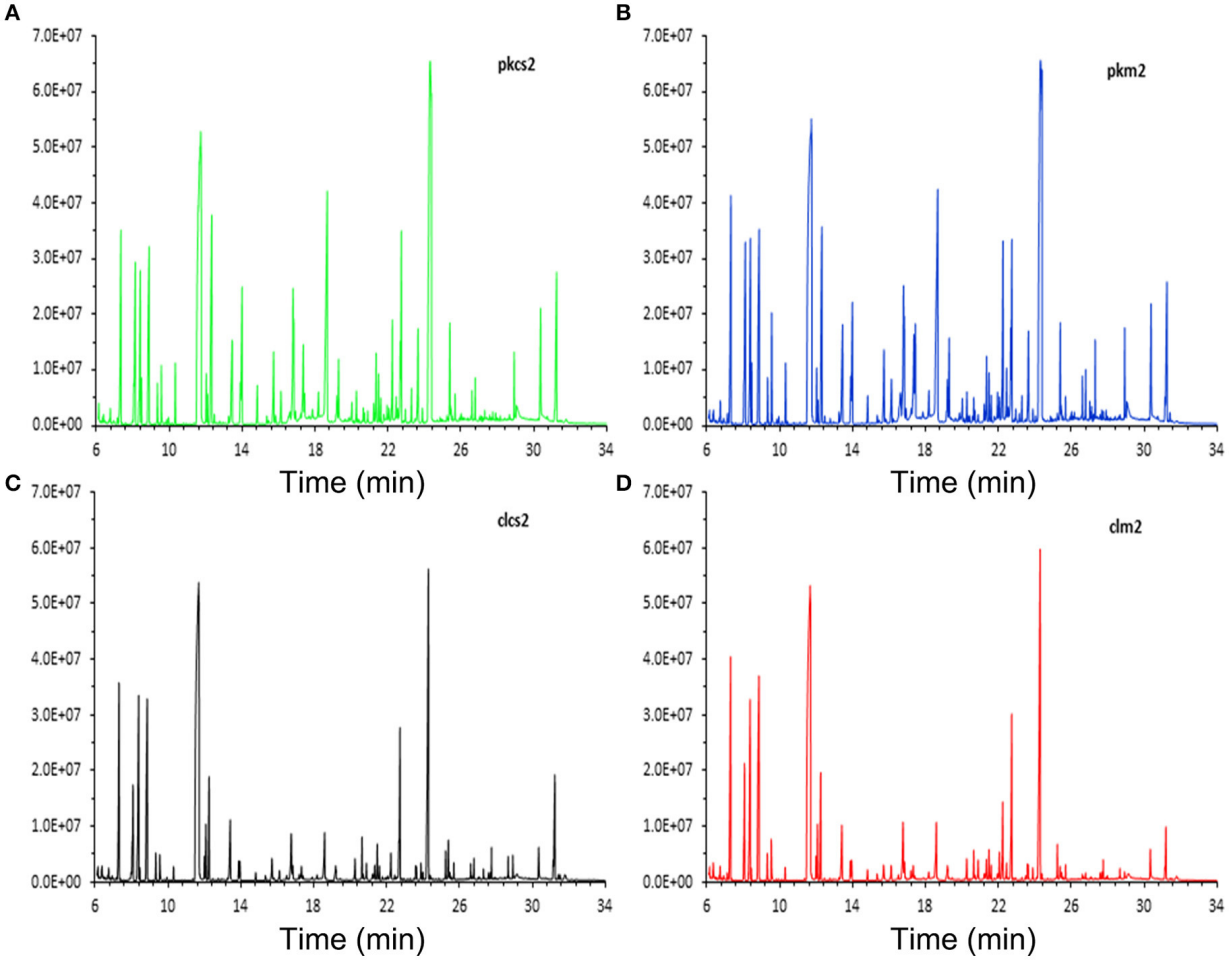
**Typical total ion chromatograms of PK-15 and 3D4/2 cell samples analyzed by GC-MS techniques. (A)** CSFV-infected PK-15 cells. **(B)** Mock-infected PK-15 cells. **(C)** CSFV-infected 3D4/2 cells. **(D)** Mock-infected 3D4/2 cells.

**Figure 3:**
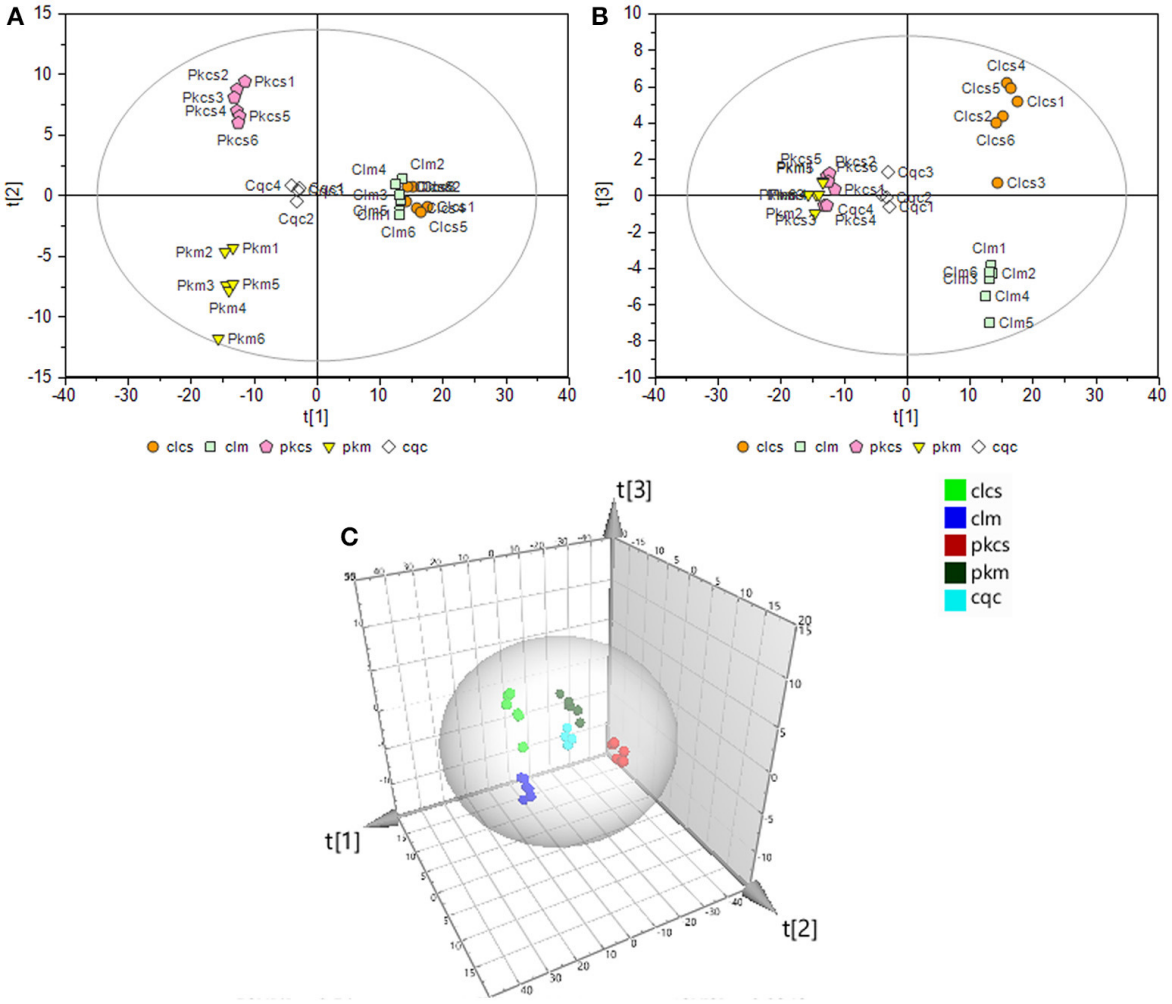
**PLS-DA of GC-MS spectra from metabolites in PK-15 (A)** and 3D4/2 **(B)** cells. **(C)** Trajectory analysis of PLS-DA score plots in the three-dimensional. In all images, pkcs and clcs represented CSFV-infected groups, pkm and clm represented mock groups, cqc represented quality control group.

The representative total ion current (TIC) chromatograms of PK-15 (Figures 2A,B) and 3D4/2 (Figures 2C,D) cell samples are shown in Figure 2. GC-MS techniques could provide more information about metabolites for the subsequent study. To visualize the metabolic variations between mock- and CSFV-infected groups, an unsupervised principal component analysis (PCA) was performed. The score plots of separate PCA demonstrated significant metabolic differences between the mock-infected group and the CSFV-infected group, whether for PK-15 cells or for 3D4/2 cells (Supplementary Figures 1A,B), suggesting that CSFV infection notably changed the metabolism of PK-15 cells and 3D4/2 cells.

To thoroughly investigate the effect of CSFV-infection on cell metabolism, a supervised partial least-squares discriminant analysis (PLS-DA) was performed on the GC-MS data of the four groups and QC samples. The first principal component (denoted as t[1] on the horizontal axis of the score plot, see Figure 3) indicates the primary variations between groups, while the second principal component (t[2] in the vertical axis of the score plots, see Figure 3) reflects the secondary differences between groups. Figure 3 shows that the metabolic difference between PK-15 and 3D4/2 cells are the main factor. The CSFV infection caused greater metabolic disturbances in PK-15 cells (see t[2] in Figure 3A) than in 3D4/2 cells (see t[3] in Figure 3B). The results suggested that CSFV infection markedly affected the metabolic profiles of host cells to varying degrees.

### Differential metabolites

To uncover the potential metabolic markers in PK-15 and 3D4/2 cells infected by CSFV, metabolites of mock- and CSFV-infected groups were statistically analyzed by combining the variable importance in the projection (VIP) values of the OPLS-DA model >1 and two-tailed Student's *t*-test (*p* < 0.05). The metabolites and fold changes (FC) of metabolites disturbed by CSFV infection in PK-15 and 3D4/2 cells are shown, respectively, in Supplementary Tables 1, 2.

The differential metabolites of PK-15 cells caused by CSFV infection are as follows: decreased glucose 6-phosphate (FC = −1.94), glyceraldehyde-3-phosphate (FC = −1.83), dihydroxyacetone phosphate (FC = −1.43), ribulose 5-phosphate (FC = −1.51), guanosine (FC = −1.24), inosine (FC = −1.16), glycerol-3-phosphate (FC = −0.68), cysteine (FC = −1.06), S-methyl-L-cysteine (FC = −1.21), cysteine sulfinic acid (FC = −1.07), and 4-hydroxyproline (FC = −1.97); and the increased 2-ketoglutaric acid (FC = 0.7), sarcosine (FC = 0.83), glycine (FC = 0.68), proline (FC = 0.62), tyrosine (FC = 0.7), 3-methyl-2-ketovaleric acid (FC = 0.7), 2-ketoisocaproic acid (FC = 0.61), and 2-ketoisovaleric acid (FC = 0.63).

On the other hand, the metabolites mostly changed by CSFV infection in 3D4/2 cells were reduced glyceraldehyde-3-phosphate (FC = −0.77), pyruvic acid (FC = −1.42), citric acid (FC = −0.44), 2-ketoglutaric acid (FC = −1.52), orotic acid (FC = −0.83), uridine 5′-monophosphate (FC = −0.51), taurine (FC = −1.12), hypotaurine (FC = −0.67), 2-ketoisocaproic acid (FC = −0.54), 3-methyl 2-ketovaleric acid (FC = −0.57), and the elevated cytosine (FC = 2.15), and cysteine sulfinic acid (FC = 1.6).

### Changed metabolic pathways

**Figure 4:**
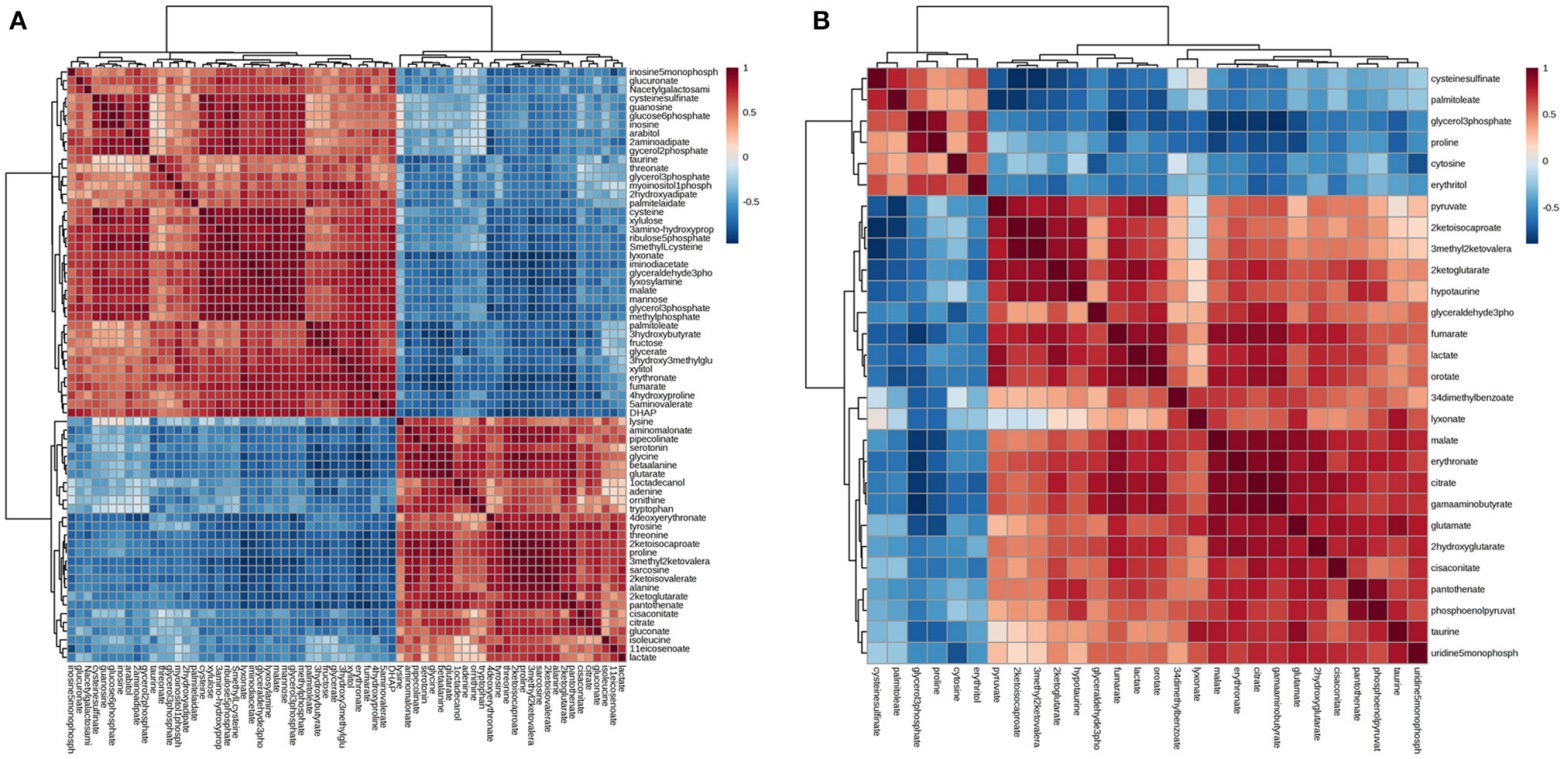
**Pearson correlation analysis plot of the differential metabolites induced by CSFV infection in PK-15 (A)** and 3D4/2 **(B)** cells.

**Figure 5:**
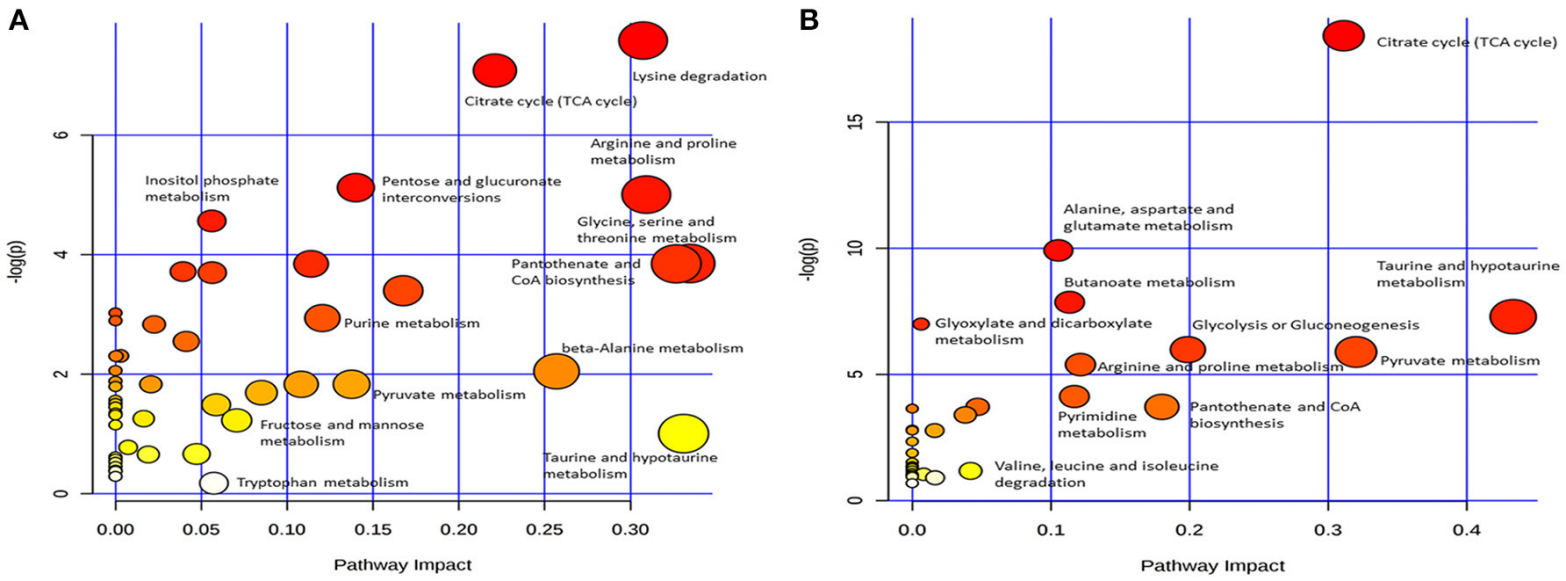
**Differentiated metabolic pathway analysis with MetaboAnalyst 2.0 in PK-15 (A)** and 3D4/2 **(B)** cells infected by CSFV.

**Figure 6:**
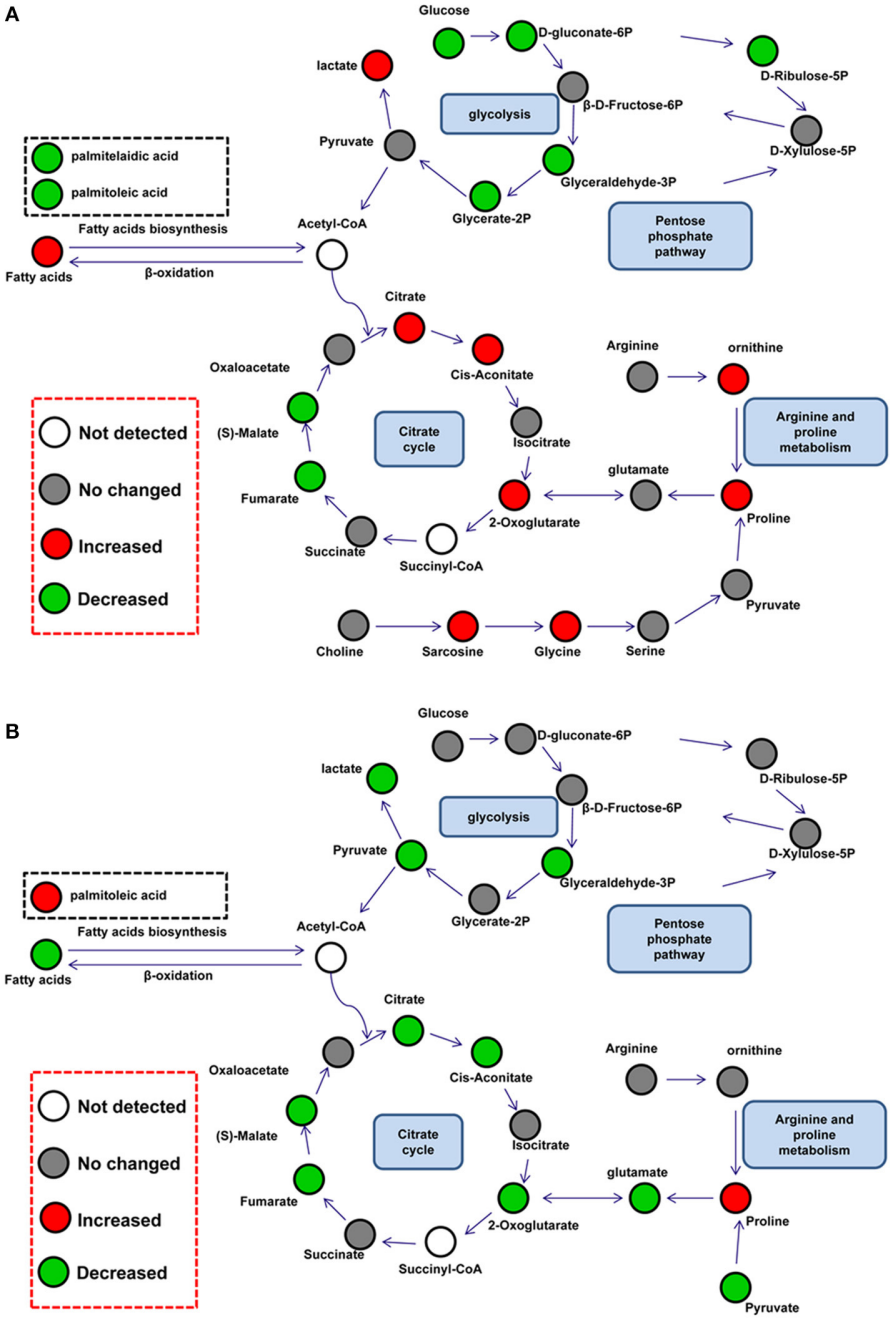
**Schematic overview of metabolic network disturbed by CSFV infection in PK-15 (A)** and 3D4/2 **(B)** cells. The metabolites are shown in different colors according to their changes: red represents increased metabolites, green represents decreased metabolites, gray represents no changed metabolites, and the white represents not detected metabolites.

Differential metabolites were further analyzed with MetaboAnalyst 2.0 to explore the effects of CSFV infection on metabolic pathways in PK-15 and 3D4/2 cells. The relationship between differential metabolites was investigated by using Pearson correlation (Figures 4A,B) and heatmap analysis (Supplementary Figures 2A,B). The results indicated that metabolites in the same or related metabolic pathways were closely connected. When the impact-value threshold of pathway analysis was set to 0.10, 13 pathways of potential change due to CSFV infection were identified in PK-15 cells (Figure 5A), in addition to the nine discovered changed pathways in 3D4/2 cells (Figure 5B). By establishing the connections between relational metabolic pathways, the metabolic networks in PK-15 (Figure 6A) and 3D4/2 (Figure 6B) cells infected with CSFV were constructed.

## Discussion

It is known that viruses appropriate energy and biosynthetic precursors from host cell metabolism for replication. Progress in metabolomics technology has now made it possible to uncover meaningful biomarkers during viral infection, which might be suitable targets for drug intervention and therapeutic vaccines (Aldridge and Rhee, 2014). Understanding the molecular mechanisms of CSF is particularly important for controlling and preventing CSF in piglets (Paton and Greiser-Wilke, 2003). In this study, for the first time, metabolomics was used to analyze changes in metabolites and metabolic pathways in CSFV-infected cells.

Several previous reports have found that viral replication promotes the citrate cycle (Vastag et al., 2011; Huang et al., 2012; Aldridge and Rhee, 2014). In PK-15 cells, CSFV infection slightly increased the levels of citric acid (FC = 0.16), cis-aconitic acid (FC = 0.18), and 2-ketoglutaric acid (FC = 0.7) in the citrate cycle, whereas it decreased levels of malic acid (FC = −0.27) and fumaric acid (FC = −0.24). From this it could be presumed that CSFV infection disturbed the citrate cycle of PK-15 cells. The decline in malic acid and fumaric acid might be the result of regulation by downstream enzymes of 2-ketoglutaric acid or be attributed to excessive transformation of 2-ketoglutaric acid to proline. Similarly, reverse levels of intermediates in the citrate cycle are also discovered in cells infected by HIV-1 or human cytomegalovirus (HCMV) (Munger et al., 2006; Liao et al., 2012). However, five pivotal intermediates of the citrate cycle: citric acid (FC = −0.44), cis-aconitic acid (FC = −0.28), 2-ketoglutaric acid (FC = −1.52), malic acid (FC = −0.38), and fumaric acid (FC = −0.31), were down-regulated in 3D4/2 cells infected with CSFV. This suggested that the citrate cycle might be inhibited by CSFV infection in 3D4/2 cells and this might be caused directly by a reduction of citrate synthesis, which is also found in T-cells treated with HIV-1 Tat protein (Liao et al., 2012).

In addition to the citrate cycle, glycolysis is also changed in CSFV-infected mammalian cells. Glycolytic intermediates showed lower levels in CSFV-infected PK-15 cells than mock-infected PK-15 cells. Previous proteomic analysis has showed that CSFV infection improves expression of glycolytic enzymes in PK-15 cells (Sun et al., 2008). Together with up-regulated lactic acid (FC = 0.09) downstream of glycolysis and citric acid (FC = 0.16) upstream of the citrate cycle, the decreased glycolytic intermediates might be caused by an increased rate of glycolysis or the citrate cycle in PK-15 cells infected with CSFV. However, decreased levels of glyceraldehyde-3P (FC = −0.77), pyrutate (FC = −1.42), lactate (FC = −0.21), and citric acid (FC = −0.44) showed that glycolysis might be inhibited by CSFV infection in 3D4/2 cells.

Increased levels of glycine (FC = 0.68), proline (FC = 0.62), and ornithine (FC = 0.56) were detected in PK-15 cells infected with CSFV. All of these amino acids can be transformed from glycolytic and citrate cycle intermediates, such as pyruvic acid, 2-ketooglutaric acid, and succinyl-CoA, to provide protein enzymes requirements in cells. Hence one possible source of these up-regulated amino acids is the conversion of intermediates of central carbon metabolism. In addition, according to our previous finding that CSFV activates RNA-dependent protein kinase (PKR) to enhance viral replication in PK-15 cells, another possible speculation is that inhibition of protein synthesis via activation of PKR reduces the utilization of amino acids (Liu et al., 2015). Notably, proline levels (FC = 0.14) in 3D4/2 cells infected by CSFV showed an increased tendency. Conversion of arginine to proline is an important pathway for regulating arginine content. Arginine is closely related to the production of nitric oxide (NO) in macrophages (Mahmoud et al., 1999), and a previous report has suggested that CSFV can inhibit antiviral function of NO in macrophages (Zaffuto et al., 2007). Here, we speculated that increased levels of proline might be converted from arginine, and that CSFV might inhibit the production of NO by regulating the content of arginine in 3D4/2 cells.

As a single-positive RNA virus, CSFV must utilize cellular precursors for nucleotide biosynthesis to accomplish viral replication (Friis et al., 2012). In PK-15 cells infected by CSFV, levels of materials in purine biosynthesis including guanosine (FC = −1.24), inosine (FC = −1.16), and inosine-5′-monophosphate (FC = −0.5) were decreased. Whereas the content of adenine (FC = 0.25) in PK-15 cells was slightly increased; these are direct substrates for ATP biosynthesis. Meanwhile, in addition to decreased levels of ribulose-5-phosphate (FC = −1.51) in the pentose phosphate pathway, the reduced content of intermediates including arabitol (FC = −0.63), glucuronic acid (FC = −0.31), xylitol (FC = −0.34) and xylulose (FC = −1.12) in the pentose and glucoronate interconversion pathways manifested consummation of pentose was promoted. According to these data, we speculated that nucleotide biosynthesis was enhanced by CSFV infection in PK-15 cells for viral replication. Similar speculation was based on increased levels of cytosine (FC = 2.15) and decreased UMP (FC = −0.51) in 3D4/2 cells infected by CSFV. Several previous reports show that the synthesis of purine and pyrimidine is improved in virus-infected cells (Munger et al., 2006; Vastag et al., 2011).

Lipid metabolism of host cells is important for the biogenesis of virus envelopes and viral budding (Lin et al., 2010; Tanner et al., 2014). In this study, palmitoleic acid (FC = −0.55) and palmitelaidic acid (FC = −0.22) relating to fatty acid metabolism, as well as glycerol-3-phosphate (FC = −0.68), glycerol-2-phosphate (FC = −0.32), and myo-inositol-1-phosphate (FC = −0.19) in glycerolipid metabolism, displayed a decreased level in PK-15 cells infected by CSFV. These data suggested that CSFV infection utilized a subset of intermediate metabolites for lipid synthesis and subsequent viral assembly in PK-15 cells. For 3D4/2 cells, however, CSFV infection increased the levels of palmitoleic acid (FC = 0.3) during fatty acid metabolism and sn-glycero-3-phospho-1-inositol (FC = 0.49) during glycerolipid metabolism. The reverse regulation of fatty acid metabolism might result from lower replication of CSFV in 3D4/2 cells compared with PK-15 cells (Pei et al., 2014).

Taurine is an amino acid analog closely connected to osmoregulation and volume control in mammalian cells. In hypo-osmotic conditions, cellular taurine is rapidly exuded to prevent cell turgidity, thus maintaining osmotic balance (Lambert, 2004). Notably, decreased levels of taurine were observed both in PK-15 (FC = −0.37) and 3D4/2 cells (FC = −1.12) infected by CSFV. This suggested that CSFV might utilize the regulation of cellular taurine to preserve cell volume and inhibit cytopathic effects.

In conclusion, our metabolomics data provided evidence that CSFV rebuilt metabolic networks in host cells. Metabolic pathways that were altered included glycolysis, the citrate cycle, nucleotide biosynthesis, amino acid metabolism, and lipid metabolism. Metabolic interplay between CSFV and host cells offers a new means of clarification of the processes of CSF. Data from the study offers possible targets for new biomarkers for diagnosis, enzyme inhibitors, or metabolites for use as antiviral drugs (Matthews et al., 2016), or screening viral gene products for vaccines (Holtfreter et al., 2016). However, potential strategies involved in the perturbation of host cell metabolism, caused by CSFV infection, have not been clarified until now. Direct modulation of metabolites or indirect modulation of enzyme concentrations are both possible. Future experiments combining transcriptomics, proteomics, and metabolomics are needed to comprehensively answer these questions. In addition, the protocols was paid attention by the reviewers that 6-well plates have been used to cultivate cells for sample preparation in some studies (Birungi et al., 2010; Hollenbaugh et al., 2011), while 10 cm dishes have been used in other studies (Munger et al., 2006; Vastag et al., 2011). There may be intrinsic biases affecting each dish (e.g., position in incubator) when 10 cm dishes are used compared to 6-well plates. Whether this is an influential factor in the metabolomics analysis, needs to be addressed by further study.

## Author contributions

HG carried out the data analysis and drafted the manuscript. HX prepared materials for the experiments. JY participated in the design of the study. MZ and JC conceived the study. HD participated in its design and coordination. All authors read and approved the final manuscript.

### Conflict of interest statement

The authors declare that the research was conducted in the absence of any commercial or financial relationships that could be construed as a potential conflict of interest.
