# Supplementary material for: Metabolic Profiles in Cell Lines Infected with Classical Swine Fever Virus
Source: Front Microbiol. 2017 Apr 20;8:691. doi: 10.3389/fmicb.2017.00691 (PMC5397473; doi:10.3389/fmicb.2017.00691)
Supplement: Supplementary file 1 [file Table1.docx]

**Metabolic Profiles in Cell Lines Infected with Classical Swine Fever Virus**

Hongchao Gou^#^, Mingqiu Zhao^#^, Jin Yuan, HailuanXu, Hongxing Ding, Jinding Chen*

College of Veterinary Medicine, South China Agricultural University, Guangzhou, People’s Republic of China

^#^These authors contributed equally to this work.

***Correspondence:**

Jinding Chen

Email: jdchen@scau.edu.cn

**1 Supplementary Figures and Tables**

**1.1 Supplementary Figures**


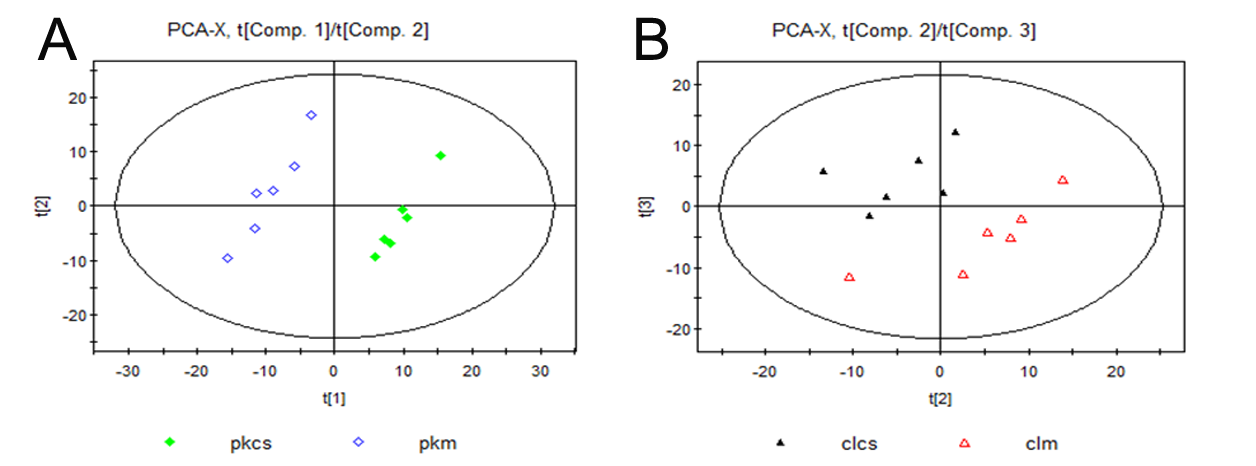


**Supplemental Figure 1.** PCA of GC-MS spectra from metabolites in PK-15 (**A**) and 3D4/2 (**E**) cells. In all images, pkcs and clcs represented CSFV-infected groups, and pkm and clm represented mock groups.


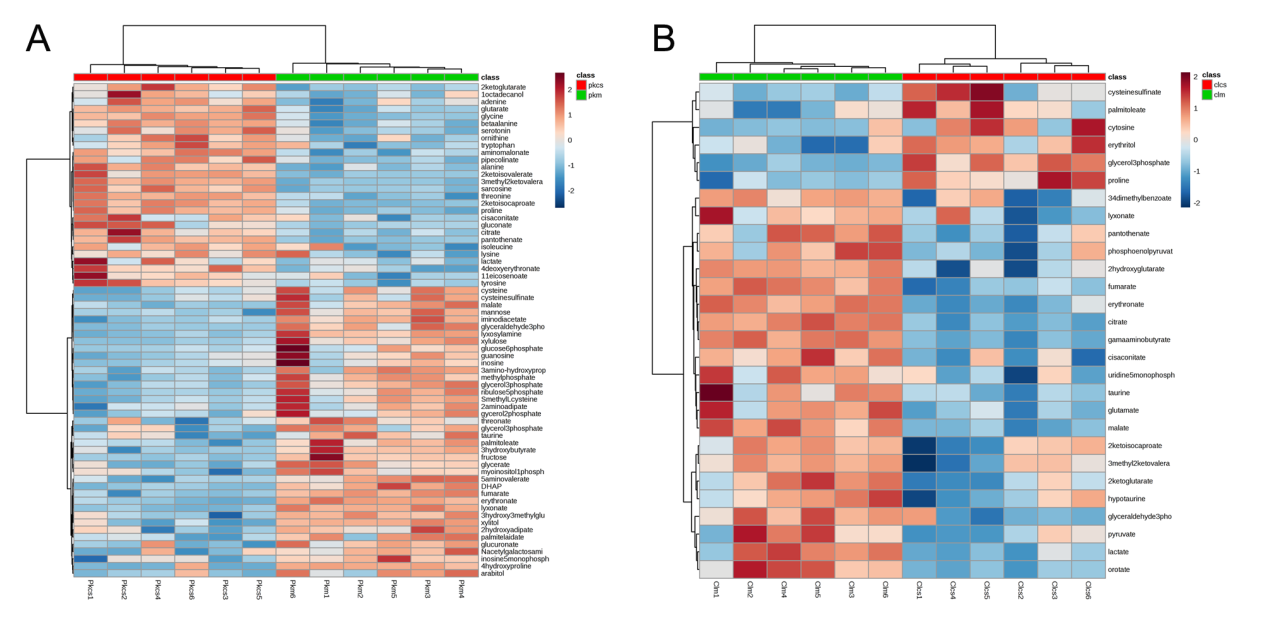


**Supplemental Figure 2.** Heatmap visualization of differentiated metabolites in PK-15 (A) and 3D4/2 (B) cells. Rows: metabolites; columns: samples.

**1.2 Supplementary Tables**

**Table 1. Identification of metabolites differentiated by CSFV infection in PK-15 cells**

| **Metabolites** | **VIP^a^** | **p-value^b^** | **FC** | **Pathway** |
| --- | --- | --- | --- | --- |
| glucose-6-phosphate | 1.13 | 2.74E-02 | -1.94 | Glycolysis / Gluconeogenesis; |
| glyceraldehyde-3-phosphate | 1.60 | 8.60E-05 | -1.83 | Glycolysis / Gluconeogenesis |
| DHAP | 1.66 | 1.29E-05 | -1.43 | Glycolysis / Gluconeogenesis |
| citric acid | 1.43 | 1.73E-03 | 0.16 | Citrate cycle (TCA cycle);  Alanine, aspartate and glutamate metabolism |
| 2-ketoglutaric acid | 1.58 | 1.28E-04 | 0.70 | Citrate cycle (TCA cycle) |
| cis-aconitic acid | 1.18 | 1.95E-02 | 0.18 | Citrate cycle (TCA cycle) |
| malic acid | 1.54 | 3.23E-04 | -0.27 | Citrate cycle (TCA cycle);  Pyruvate metabolism |
| fumaric acid | 1.70 | 1.73E-06 | -0.24 | Citrate cycle(TCA cycle);  Alanine,aspartate and glutamate metabolism |
| alanine | 1.63 | 3.94E-05 | 0.22 | Alanine, aspartate and glutamate metabolism;  Cysteine and methionine metabolism;  Taurine and hypotaurine metabolism |
| beta-alanine | 1.60 | 7.90E-05 | 0.51 | Alanine and aspartate metabolism |
| gluconic acid | 1.35 | 4.39E-03 | 0.15 | Pentose phosphate pathway |
| ribulose-5-phosphate | 1.49 | 7.18E-04 | -1.51 | Pentose phosphate pathway |
| glyceric acid | 1.50 | 6.13E-04 | -0.78 | Pentose phosphate pathway;  Glycine,serine and threonine metabolism;  Glycerolipid metabolism |
| sarcosine | 1.67 | 9.48E-06 | 0.83 | Glycine, serine and threonine metabolism |
| cysteine | 1.38 | 3.34E-03 | -1.06 | Glycine, serine and threonine metabolism; Cysteine and methionine metabolism;  Taurine and hypotaurine metabolism |
| glycine | 1.69 | 2.90E-06 | 0.68 | Glycine,serine and threonine metabolism;  Lysine degradation |
| threonine | 1.68 | 4.85E-06 | 0.34 | Glycine,serine and threonine metabolism |
| 4-hydroxyproline | 1.51 | 5.61E-04 | -1.97 | Arginine and proline metabolism |
| ornithine | 1.26 | 1.08E-02 | 0.56 | Arginine and proline metabolism |
| proline | 1.68 | 6.59E-06 | 0.62 | Arginine and proline metabolism |
| glutaric acid | 1.62 | 4.51E-05 | 0.34 | Fatty acid degradation;  Lysine degradation |
| 2-aminoadipic acid | 1.20 | 1.74E-02 | -0.22 | Fatty acid, monoamino |
| 2-hydroxyadipic acid | 1.28 | 8.96E-03 | -0.18 | Fatty acid, monohydroxy |
| 1-octadecanol | 1.22 | 1.51E-02 | 0.21 | Fatty alcohol |
| 11-eicosenoic acid | 1.16 | 2.27E-02 | 0.31 | Fatty acid metabolism |
| palmitelaidic acid | 1.33 | 5.76E-03 | -0.22 | Fatty acid metabolism |
| palmitoleic acid | 1.48 | 8.57E-04 | -0.55 | Fatty acid metabolism |
| 5-aminovaleric acid | 1.53 | 3.68E-04 | -0.33 | Lysine degradation |
| pipecolinic acid | 1.51 | 5.70E-04 | 0.30 | Lysine degradation |
| lysine | 1.06 | 4.16E-02 | 0.30 | Lysine degradation;  Biotin metabolism |
| arabitol | 1.21 | 1.58E-02 | -0.63 | Pentose and glucoronate interconversions |
| glucuronic acid | 1.29 | 7.82E-03 | -0.31 | Pentose and glucoronate interconversions |
| xylitol | 1.59 | 1.10E-04 | -0.34 | Pentose and glucoronate interconversions |
| xylulose | 1.49 | 7.12E-04 | -1.12 | Pentose and glucoronate interconversions |
| adenine | 1.32 | 6.00E-03 | 0.25 | Purine metabolism |
| guanosine | 1.18 | 1.97E-02 | -1.24 | Purine metabolism |
| inosine | 1.06 | 4.26E-02 | -1.16 | Purine metabolism |
| inosine-5'-monophosphate | 1.25 | 1.10E-02 | -0.50 | Purine metabolism |
| serotonin | 1.49 | 7.93E-04 | 0.61 | Tryptophan metabolism |
| tryptophan | 1.28 | 9.22E-03 | 0.23 | Tryptophan metabolism;  Glycine, serine and threonine metabolism |
| tyrosine | 1.47 | 1.10E-03 | 0.70 | Tyrosine metabolism;  Phenylalanine metabolism |
| isoleucine | 1.09 | 3.59E-02 | 0.16 | Valine, leucine and isoleucine degradation |
| 3-methyl-2-ketovaleric acid | 1.75 | 6.25E-08 | 0.70 | Valine, leucine and isoleucine degradation |
| 2-ketoisocaproic acid | 1.73 | 3.63E-07 | 0.61 | Valine, leucine and isoleucine degradation |
| 2-ketoisovaleric acid | 1.66 | 1.48E-05 | 0.63 | Valine, leucine and isoleucine degradation |
| threonic acid | 1.17 | 2.08E-02 | -0.22 | Ascorbate and aldarate metabolism |
| myo-inositol-1-phosphate | 1.29 | 8.00E-03 | -0.19 | Inositol phosphate metabolism  Glycerolipid metabolism;  Glycerophospholipid metabolism |
| 3-hydroxybutyric acid | 1.52 | 4.40E-04 | -0.18 | ketone bodies |
| glycerol-3-phosphate | 1.60 | 8.28E-05 | -0.68 | Glycerolipid metabolism |
| glycerol-2-phosphate | 1.26 | 1.05E-02 | -0.32 | Glycerolipid metabolism |
| sn-glycero-3-phospho-1-inositol | 1.22 | 1.44E-02 | -0.21 | Glycerolipid metabolism;  Glycerophospholipid metabolism |
| fructose | 1.51 | 5.76E-04 | -0.90 | Starch and sucrose metabolism;  Amino sugar and nucleotide sugar metabolism |
| taurine | 1.25 | 1.16E-02 | -0.37 | Taurine and hypotaurine metabolism |
| pantothenic acid | 1.70 | 2.33E-06 | 0.45 | beta-Alanine metabolism;  Pantothenate and CoA biosynthesis |
| mannose | 1.56 | 2.10E-04 | -0.99 | Fructose and mannose metabolism;  Amino sugar and nucleotide sugar metabolism |
| lactic acid | 1.07 | 3.94E-02 | 0.09 | Pyruvate metabolism |
| lyxonic acid | 1.75 | 6.22E-08 | -0.84 | a sugar acid |
| 3-amino-2-hydroxypropionic acid | 1.44 | 1.63E-03 | -0.54 |  |
| 3-hydroxy-3-methylglutaric acid | 1.51 | 5.39E-04 | -0.42 |  |
| 4-deoxyerythronic acid | 1.45 | 1.41E-03 | 0.28 |  |
| aminomalonic acid | 1.61 | 7.25E-05 | 0.50 |  |
| cysteine sulfinic acid | 1.14 | 2.53E-02 | -1.07 | Cysteine and methionine metabolism; Taurine and hypotaurine metabolism |
| erythronic acid | 1.74 | 1.29E-07 | -0.43 |  |
| iminodiacetic acid | 1.64 | 3.11E-05 | -0.44 |  |
| lyxosylamine | 1.66 | 1.57E-05 | -1.16 |  |
| methylphosphate | 1.56 | 2.35E-04 | -0.61 |  |
| N-acetylgalactosamine | 1.20 | 1.70E-02 | -1.13 |  |
| S-methyl-L-cysteine | 1.43 | 1.90E-03 | -1.21 |  |

^a^Variable importance in the projection (VIP) values were obtained from the OPLS-DA model.

^b^The *p* value was calculated from two-tailed Student’s *t* test.

^c^Fold change (FC) was calculated as a binary logarithm of the average mass response (normalized peak area) ratio between CSFV-infected groups vs mock groups, where a positive value means that the average mass response of the metabolite in CSFV-infected groups is larger than that in mock groups.

**Table 2. Identification of metabolites differentiated by CSFV infection in 3D4/2 cells**

| **Metabolites** | **VIP^a^** | **p-value^b^** | **FC^c^** | **Pathway** |
| --- | --- | --- | --- | --- |
| 2-ketoglutaric acid | 1.84 | 3.76E-03 | -1.52 | Citrate cycle (TCA cycle) |
| cis-aconitic acid | 1.83 | 4.25E-03 | -0.28 | Citrate cycle (TCA cycle) |
| citric acid | 2.35 | 8.65E-08 | -0.44 | Citrate cycle (TCA cycle);  Alanine, aspartate and glutamate metabolism |
| malic acid | 2.27 | 4.63E-06 | -0.38 | Citrate cycle (TCA cycle);  Pyruvate metabolism |
| fumaric acid | 2.24 | 1.24E-05 | -0.31 | Citrate cycle (TCA cycle);  Alanine, aspartate and glutamate metabolism |
| glyceraldehyde-3-phosphate | 1.89 | 2.48E-03 | -0.77 | Glycolysis / Gluconeogenesis |
| phosphoenolpyruvic acid | 1.66 | 1.35E-02 | -0.32 | Glycolysis / Gluconeogenesis |
| pyruvic acid | 1.50 | 3.08E-02 | -1.42 | Glycolysis/Gluconeogenesis;  Citrate cycle (TCA cycle);  Pentose phosphate pathway |
| gamma-aminobutyric acid | 2.36 | 2.74E-08 | -0.54 | Alanine, aspartate and glutamate metabolism; Arginine and proline metabolism;  beta-Alanine metabolism |
| glutamic acid | 2.02 | 6.69E-04 | -0.31 | Alanine, aspartate and glutamate metabolism; Arginine and proline metabolism;  Taurine and hypotaurine metabolism |
| proline | 1.98 | 1.01E-03 | 0.14 | Arginine and proline metabolism |
| pantothenic acid | 1.73 | 8.71E-03 | -0.20 | beta-Alanine metabolism;  Pantothenate and CoA biosynthesis |
| palmitoleic acid | 1.50 | 3.04E-02 | 0.30 | Fatty acid metabolism |
| cytosine | 1.46 | 3.76E-02 | 2.15 | Pyrimidine metabolism |
| orotic acid | 2.13 | 1.46E-04 | -0.83 | Pyrimidine metabolism |
| uridine-5'-monophosphate | 1.65 | 1.41E-02 | -0.51 | Pyrimidine metabolism |
| lactic acid | 1.98 | 1.01E-03 | -0.21 | Pyruvate metabolism |
| hypotaurine | 1.54 | 2.49E-02 | -0.67 | Taurine and hypotaurine metabolism |
| taurine | 1.81 | 4.85E-03 | -1.12 | Taurine and hypotaurine metabolism |
| 3-methyl-2-ketovaleric acid | 1.56 | 2.30E-02 | -0.57 | Valine, leucine and isoleucine degradation |
| 2-ketoisocaproic acid | 1.41 | 4.50E-02 | -0.54 | Valine, leucine and isoleucine degradation |
| sn-glycero-3-phospho-1-inositol | 2.25 | 9.49E-06 | 0.49 | Glycerolipid metabolism;  Glycerophospholipid metabolism |
| 2-hydroxyglutaric acid | 1.96 | 1.31E-03 | -0.23 |  |
| 3,4-dimethylbenzoic acid | 1.63 | 1.61E-02 | -0.94 |  |
| cysteine sulfinic acid | 1.53 | 2.59E-02 | 1.60 |  |
| erythritol | 1.67 | 1.23E-02 | 0.22 |  |
| erythronic acid | 2.27 | 4.63E-06 | -0.33 |  |
| lyxonic acid | 1.45 | 3.81E-02 | -0.22 |  |

^a^Variable importance in the projection (VIP) values were obtained from the OPLS-DA model.

^b^The *p* value was calculated from two-tailed Student’s *t* test.

^c^Fold change (FC) was calculated as a binary logarithm of the average mass response (normalized peak area) ratio between CSFV-infected groups vs mock groups, where a positive value means that the average mass response of the metabolite in CSFV-infected groups is larger than that in mock groups.
